# Histogen Layers Contributing to Adventitious Bud Formation Are Determined by their Cell Division Activities

**DOI:** 10.3389/fpls.2017.01749

**Published:** 2017-10-17

**Authors:** Tomoyuki Nabeshima, Soo-Jung Yang, Sho Ohno, Keita Honda, Ayumi Deguchi, Motoaki Doi, Fumi Tatsuzawa, Munetaka Hosokawa

**Affiliations:** ^1^Laboratory of Vegetable and Ornamental Horticulture, Graduate School of Agriculture, Kyoto University, Kyoto, Japan; ^2^Laboratory of Vegetable and Ornamental Horticulture, Faculty of Agriculture, Iwate University, Morioka, Japan

**Keywords:** periclinal chimera, *Saintpaulia*, true-to-type propagation, adventitious shoot, epidermis, pinwheel, anthocyanin, color pattern

## Abstract

*Saintpaulia ionantha* is propagated by adventitious buds in horticulture, and periclinal chimeral cultivars are usually difficult to propagate. However, some periclinal chimeral cultivars can be propagated with adventitious buds, and the mechanism of which has been unknown. Striped flower cultivars “Kaname,” “Concord,” and “Monique” were used to investigate what causes flower color separation in adventitious shoot-derived plants by tissue culture. These cultivars were revealed to have mutated *flavonoid 3*′*, 5*′ *hydroxylase* (*SiF3*′*5*′*H*), *WDR1* (*SiWDR1*), or *flavonoid 3 hydroxylase* (*SiF3H*), respectively, in their L1 layer. From our previous study using “Kaname,” all flowers from adventitious shoots were colored pink, which was the epidermal color of mother plants' flowers. We used “Concrd” and “Monique” from which we obtained not only monochromatic-colored plants the same as the epidermal color of mother plants, but also plants with a monochromatic colored plants, same as the subepidermal color, and a striped flower color the same as mother plants. Histological observations revealed that epidermal cells divided actively at 14 d after culture and they were involved in the formation of adventitious shoots in the cultured leaf segments of “Kaname.” On the other hand, in “Concord” and “Monique,” the number of divided cells in the subepidermis was rather higher than that of epidermal cells, and subepidermal cells were sometimes involved in shoot formation. In addition, the plant and leaf size of L1-derived plants from “Concord” and “Monique” were non-vigorous and smaller than those derived from the subepidermal layer. In conclusion, periclinal chimeral cultivars of *Saintpaulia* can be divided into two types. One type has a high cell division activity in the L1 layer, from which only single flower-colored plants derived from L1 can be obtained as adventitious shoots. Another type has a low cell division activity in the L1 layer, from which striped flower-colored plants the same as mother plants derived from several layers including L1 can be obtained as adventitious shoots. In the periclinal chimeral cultivar capable of propagation with adventitious shoots, the possibility was shown that cells in the L2 layer could form shoots by involving cells of the L1 layer with a low division activity.

## Introduction

In *Saintpaulia ionantha*, colloquially known as the African violet, adventitious shoots can be easily induced from most organs by tissue culture and therefore, it has been used as a model plant for studying adventitious shoot formation (Naylor and Johnson, 1937; Arisumi and Frazier, 1968; Kukulczanka and Suszynska, 1972; Vazquez and Short, 1978; Ohki, 1994; Nielsen et al., 2003; Yang et al., 2017). *Saintpaulia* cultivars include many variegated phenotypes in their flowers, such as white-edged leaves or striped petals. In these variegated cultivars, micropropagation, which include adventitious shoot induction by tissue culture techniques, often produce plants with monochromatic or variegated patterns different from their mother plants. These phenomena can be explained by periclinal chimeral separation.

The aerial parts of all vascular plants are composed of cells generated from a shoot apical meristem (SAM). Angiosperm SAMs are composed of several cell layers called L1, L2, and L3 that are numbered from the outermost layer (Satina et al., 1940). When cell layers with different genetic backgrounds compose a SAM, this plant is called a periclinal chimera. Basically, cell divisions in L1 and L2 layers are strictly anticlinal in direction and those in the L3 layer are random (Stewart and Dermen, 1975). Stable periclinal chimeras can retain their SAM structure through their growth and a variety of color patterns appear in their petals, which are composed by L1 and L2 layers in *Arabidopsis* (Jenik and Irish, 2000). In general, adventitious shoot formation is thought to occur from a single cell in many plants (Broertjes et al., 1968; Broertjes and Keen, 1980; Abu-Qaoud et al., 1990). Histological observations at adventitious shoot formation from leaves in *Saintpaulia* also suggested that only their epidermal layer participates in new SAM formation (Naylor and Johnson, 1937; Broertjes et al., 1968; Broertjes and van Harten, 1985; Peary et al., 1988; Ohki, 1994; Lo, 1997; Hosokawa et al., 1998). SAM of this single layer originate adventitious buds that must be composed by a single genetic background, and thus their phenotypes often differ from the mother plant, whose SAM is composed by several genetically distinct cell layers. However, there are some exceptions to this rule because in some cases true-to-type patterned plants occurred in adventitious shoots from variegated mother plants (Lineberger and Druckenbrod, 1985; Ando et al., 1986; Peary et al., 1988; Sandall and Lineberger, 1997; Nielsen et al., 2003), and therefore multiple layer contributions by regeneration have to be supposed in these plants. We should note that a part of these observations mislead the conclusions, because plant materials without evidence of a periclinal chimeral structure were used in the studies. For example, Norris et al. (1983) used variegated cultivars “Tommie Lou,” “Bold Dance,” “Marge Winters,” and “Calico Kitten,” and based on their observation that all regenerates from these cultivars showed a true-to-type pattern, they suggested the multi-cellar organization in the ontogenesis of adventitious buds in some cultivars. However, subsequent reports suggested that the variegation patterns in “Tommy Lou” mother plants were not the results of chimeral structure in their SAMs, but their variegation patterns were controlled by genetic elements carried by a single genotype (Marcotrigiano et al., 1984). In this case, analysis of variegated pattern in the regenerants would not make sense for determination of the origin of adventitious buds, and such genetic control of variegated patterns was seen in many cultivars of *Saintpaulia* (Marcotrigiano et al., 1984). Thus, we have to determine whether a cultivar is periclinal chimera or not only from the visible phenotypes but also by genetic background in each histogen layer of mother plants. Nevertheless, sequential debates on “Tommie Lou” type variegations and possible existences of other types of non-chimeral variegated plants, do not deny the entire previously reported multi-cell layer-derived regenerations. Naylor and Johnson (1937) reported that not only epidermal but also subepidermal cells were actively divided in explants of petiole and lamina during tissue culturing. Based on time-course observations on adventitious formation from petioles, they noted that “Although the shoot has its origin in a single epidermal cell, adjacent epidermal cells and parenchyma cells within the petiole contribute to its final formation” (Naylor and Johnson, 1937). Furthermore, the case of shoot regeneration from a synthetic generated chimera using different *Nicotiana* species, shoot regeneration with reorganized structure of the shoot apical meristem sometimes occurs, which indicated multi-cell participation for shoot regeneration (Marcotrigiano and Gouin, 1984a,b; Marcotrigiano, 1986). Espino and Vazquez (1981) observed that cytochimeras, which was mosaic of cells with different ploidy levels, could be obtained by colchicine treatment followed by adventitious bud induction. These reports strongly suggest the multiple cell layer contribution for adventitious bud organogenesis in some cases, however, the factors that determine true-to-type regeneration from chimeral plants are not understood.

To address this question, we decided to revisit a histological analysis on adventitious shoot formation using chimeral *Saintpaulia* cultivars with rigorous evidences that they were putative chimeras. We selected three cultivars, “Kaname,” “Concord,” and “Monique.” “Kaname” has pink petals with a blue stripe in the center. Our previous study indicated that the pink petal portion of “Kaname” which was derived from the L1 layer, had a mutated nonfunctional *flavonoid 3*′*, 5*′ *hydroxylase* (*SiF3*′*5*′*H*) on their genome, while the inner layer had functional *SiF3*′*5*′*H*. All of their adventitious buds produced monochromatic pink flowers, indicating that only the epidermis layer contributed to adventitious bud ontogenesis in this cultivar (Yang et al., 2017). This type of chimera separation was consistent with many other studies (Naylor and Johnson, 1937; Broertjes et al., 1968; Broertjes and van Harten, 1985; Peary et al., 1988; Ohki, 1994; Lo, 1997; Hosokawa et al., 1998). On the other hand, we selected “Concord” and “Monique,” in which a true-to-type pinwheel can be propagated at a relatively high frequency even through adventitious shoot regeneration. These two cultivars were considered to be ideal materials for investigating factors affecting a multi-cell layer contribution to adventitious shoot formation. In this study, we confirmed that “Concord” and “Monique” had periclinal chimera structures by analyzing their genetic background. Then, we compared their flower color separations and adventitious shoot formation histology to those of “Kaname.” Here, we propose that cell division activities in the epidermal layer, which is determined by the genotype of the layer, are strongly correlated with ontogenesis of true-to type chimeral shoot regeneration.

## Materials and methods

### Plant material

**Figure 1:**
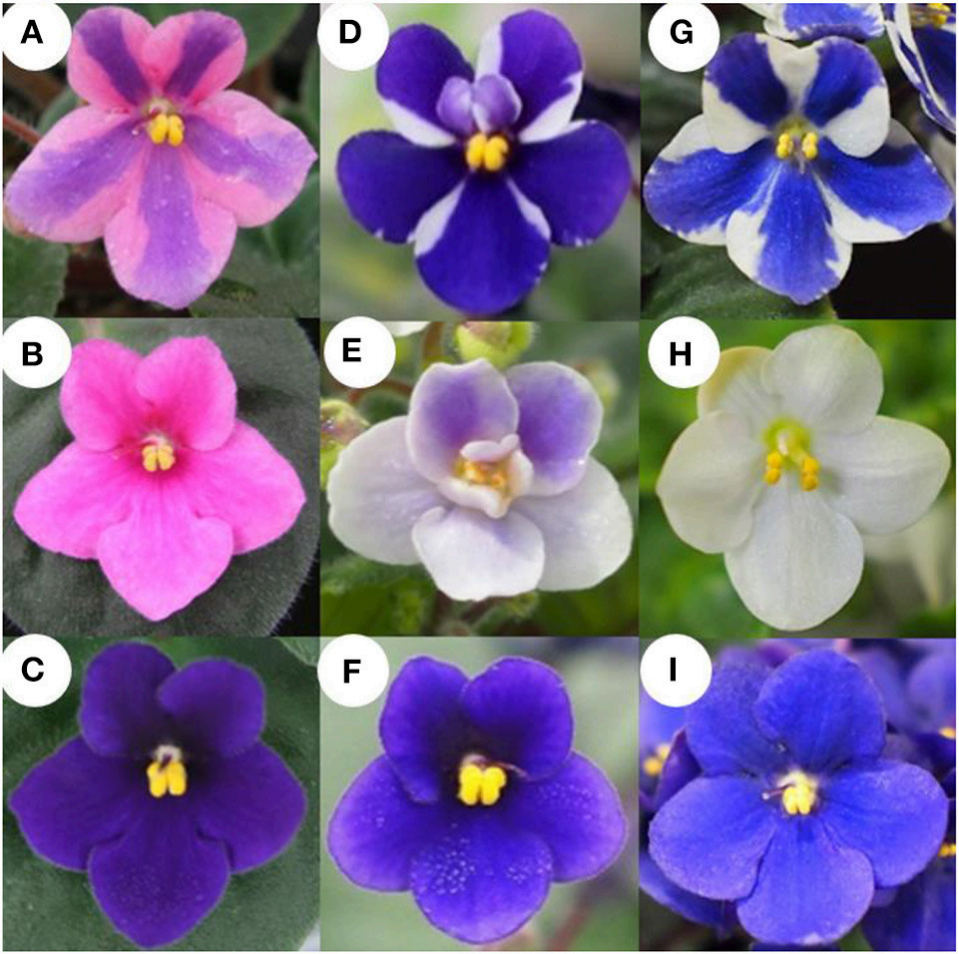
Flower phenotype of “Kaname,” “Concord,” and “Monique” and monochromatic mutants from adventitious shoots. **(A,D,G)** Original type of “Kaname,” “Concord,” and “Monique,” respectively. **(B,E,H)** Monochromatic-colored mutants whose colors are the same as epidermal layer of “Kaname,” “Concord,” and “Monique,” respectively. **(C,F,I)** Monochromatic-colored mutants whose colors are the same as subepidermal layer of “Kaname,” “Concord,” and “Monique,” respectively.

*Saintpaulia* stripe cultivars “Kaname,” “Concord,” and “Monique” were used (Figures 1A,D,G). “Kaname” has pink petals with a blue stripe in their center and “Concord” and “Monique” have white petals with blue stripes in their center. Mother plants and *in vitro*-regenerated plants were transplanted into 270 ml plastic pots containing mixed soil, vermiculite: peat moss: perlite = 5: 2: 3 (v/v/v), the pH of which was adjusted to 6–7 with dolomite. The light intensity of the greenhouse was controlled at around 250 μmol m^−2^ s^−1^ or less by covering with cheesecloths. The greenhouse temperature was maintained within 17°C–35°C by window ventilation and heating. Irrigation was carried out by water mist for 5 min at 9:00 a.m. at a rate of about 50 ml water per pot. Every other week, 50 ml of fertilizer (NPK = 6-10-5, Hyponex stock, Hyponex Japan, Japan) at 1/500 dilution was given while the temperature was adjusted to about 20°C to avoid a rapid leaf temperature drop which induces injury (Maekawa et al., 1987; Yun et al., 1998; Yang et al., 2001). Also, once in 2 months, 2–3 grains of fertilizer IB Kasei (NPK = 10-10-10, Jaycam Agri Co., Ltd., Japan) was added to each pot.

### Induction of regenerated plants from striped cultivars

Leaf blades of “Concord” and “Monique” mother plants were sterilized for 30 min with sodium hypochlorite (0.5% active chlorine) at around 20°C to avoid a rapid leaf temperature drop. Leaves were rinsed three times with 20°C sterile distilled water and cut into 1 × 1 cm squares with a sterile razor blade. They were plated on modified Murashige and Skoog (MS) (1962) medium containing benzyladenine (BA) 0.5 ppm, naphthalene acetic acid (NAA) 0.1 ppm, sucrose 3%, and solidified with 0.3% gellan gum. The pH of the medium was adjusted to 5.8 before autoclaving and after autoclaving the medium was poured into sterilized plastic dishes (9 cm ϕ). Culture was conducted in the plastic petri dishes of 9 cm in diameter and they were incubated under a 16 h day-length provided by cool-white fluorescent tubes (Lifeline, Daylight-type, NEC, Japan) with 60 μmol m^−2^ s^−1^ at 25°C. Clusters of adventitious shoots were divided into small pieces and subcultured on hormone-free modified MS medium until each shoot developed into a 5 cm leaf length. In addition, to obtain adventitious shoots from inner tissues for obtaining shoots without L1, internal tissues of the petioles were excised with a razor and tweezers and cultured in the same manner. The adventitious shoots were transplanted and rooted in a plug tray containing the previously described mixed soil and placed in a greenhouse under high-humidity by intermittent mist treatments. The shoots were then transplanted to 270 ml plastic pots and placed in the temperature and light controlled greenhouse and grown under the same irrigation and fertilization conditions as mentioned previously. When the plants flowered, the number of plants with each flower color and pattern was counted. Furthermore, to investigate the phenotype stability of these regenerated plants, leaf discs of the flowered plants for each phenotype were cultured again and the shoots were grown in the same manner. For flowered plants, each flower color phenotype of all cultivars, including “Kaname” which were cultured at the same time, measurements of plant diameter, largest leaf width, and largest leaf length were taken. Six plants of each flower color phenotype and mother plants (except for the mother plants of “Kaname”) from the three cultivars were used for the measurements. The statistical analyses used the multiple comparison test of Tukey-Kramer (*P* = 0.05) for “Concord,” and “Monique,” or a Student's *t*-test for “Kaname” (*P* = 0.05).

### Histological observation

To observe cell division and SAM formation on the cultured leaf segments of “Kaname,” “Concord,” and “Monique,” the cultured samples were fixed in a formalin-acetic acid-alcohol (FAA) solution (ethanol: formaldehyde: acetic acid: water = 12:1:1:6 v/v/v/v) after 0–18 d of culture and evacuated for several days in FAA. Then, the samples were washed with tap water and dehydrated through a series of different ethanol concentrations. Ethanol in the samples was replaced by a resin solution (Technovit 7100, Heraeus Kulzer, Germany) and then embedded in Technovit 7100 at 25°C according to the manufacturer's instructions. The samples were cut into 5 μm-thick sections using a microtome (RM2155, Leica Microsystems, Germany) and the sections were placed onto glass slides. Sections were then stained with 0.05% (w/v) toluidine blue and observed under a VHX digital microscope (Keyence, Japan). The number of cells and total cell number were counted in each epidermal layer and subepidermal layer within seven 2 × 2 mm randomly selected sections 0–14 d after culture for the three selected samples.

### High performance liquid chromatography (HPLC) analysis

Freeze-dried petals were soaked overnight in a methanol-acetic acid-water solution (methanol: acetic acid: water, 4:1:5 v/v/v) to extract the pigments. HPLC analysis was performed using an LC10A system (Shimadzu Co. Ltd., Japan) with a C18 column (Nihon Waters K.K., Japan) maintained at 40°C and a photodiode array detector. The detection wavelength was 530 and 350 nm for anthocyanins and flavones, respectively. Elutant A was 1.5% phosphate dissolved in water and elutant B was 1.5% phosphate, 20% acetic acid, and 25% acetonitrile dissolved in water. The analysis was performed at a flow rate of 1 ml/min and a column temperature of 40°C using a mobile phase gradient starting at 20–85% B over 40 min with 5 min re-equilibration at 20% B. Nine purified *Saintpaulia* pigments were used as anthocyanin standards (Tatsuzawa and Hosokawa, 2016, Table 1). To estimate the stopped position of the anthocyanin biosynthesis pathway in “Concord” and “Monique,” naringenin or taxifolin was fed to the white petals. One milli mole of naringenin (Sigma-Aldrich, USA) or taxifolin, also known as dihydroquercetin (Sigma-Aldrich), was dissolved in 1% ethanol and applied to the white edges of the petals. Distilled water containing 1% ethanol was used as a control. Just opened petals cut in the middle portion vertically to the midrib were soaked in the solution and incubated at 25°C for 24 h under the light conditions. When the petals were colored, the colored part was cut with a razor and extracted with the same solvent and analyzed by HPLC in order to determine the type of anthocyanin.

**Table 1:** The main anthocyanins and flavons detetced in each mutant.

**Cultivars, mutant**	**Anthocyanins detected by 530 nm**									**Flavons detected by 350 nm**	
	**Pg 3R5G (14.9)^a^**	**Pn 3R5G (16.8)**	**Mv 3R5G (18.0)**	**Pg 3AcR5G (21.4)**	**Pn 3AcR5G (22.6)**	**Mv 3AcR5G (23.1)**	**Cy 3AcR (24.1)**	**Pg 3AcR (26.8)**	**Pn 3AcR (28.1)**	**Lt 4′Glucuro^b^ (29.0)**	**Ap 4′Glucuro (29.4)**
Concord blue	0.4	0.3	1.1	0.1	5.8	81.7	0.6	–	–	1.9	39.4
Concord white	2.1	–	0.7	–	6.2	78.0	–	–	–	4.4	36.9
Monique blue	0.1	0.1	0.9	0.1	4.6	89.2	0.1	0.1	0.1	2.9	45.4
Monique white	–	–	0.7	0.1	1.6	82.0	0.1	0.9	–	4.6	48.2

*Pg, pelargonidin; Pn, peonidin; Mv, malvidin; R, rutinose; G, glucose; Ac, acetic acid; Lt, luteolin; Ap, apigenin; Glucuro, glucuronic acid*.

*All pigments were extratcted with MAW*.

a *indicates retention time (min)*.

b *The structure of Lt 4′-Glucuro (Luteolin 4′-glucuronide) purified from dried mixed flowers (over 10 cvs) (20 g) was confirmed on the analysis of its ^1^H (400 MHz), ^13^C (100 MHz), and 2D (COSY, NOESY, ^1^H-^13^C HMQC, and ^1^H-^13^C HMBC) NMR (JMN AL-400, JEOL Ltd., Japan) spectra in DMSO-d_6_, as well as their high resolution fast atom bombardment mass spectra (HR-FABMS) (LMS-700, JEOL, Ltd.). The ^1^H NMR spectrum of “Lt 4′-Glucuro” exhibited six aromatic proton signals of luteolin (see below) and five signals of glucuronic acid. The anomeric proton signal of glucuronic acid was observed at 5.10 (d, J = 7.1 Hz). The bonding of these compounds was confirmed by NOESY and ^1^H-^13^C HMBC experiment. The NOEs between H-1 (5.10) of glucuronic acid and H-5′ (7.18) of luteolin were observed. Moreover, the signal of the anomeric proton of glucuronic acid correlated with that of the C-4′ (148.2) carbon of luteolin in the HMBC spectrum. These results suggested that OH-4′ of luteolin was bonded with glucuronic acid. Therefore, the structure was elucidated to be luteolin 4′-glucronide*.

*Luteolin 4′-glucronide: UV-Vis (λmax nm): MeOH 333,287sh,268, +NaOMe 353,317,273, +AlCl_3_ 375sh,352,291sh,277,252sh, +AlCl_3_/HCl 376sh,346,289sh,280,247sh, +NaOAc 333, 271, +NaOAc/H3BO3 334,270. HR-FABMS [M+H]^+^, calc. for C_21_H_19_O_12_: 463.0877. found: 463.0878. 1H NMR: d luteolin: 6.80 (brs, H-3), 6.20 (d, J = 2.0 Hz, H-6), 6.84 (d, J = 2.0 Hz, H-8), 7.49 (d, J = 2.2 Hz, H-2'), 7.18 (d, J = 8.8 Hz, H-5′), 7.52 (dd, J = 2.2, 8.8 Hz, H-6′), glucuronide: 5.10 (d, J = 7.1 Hz, H-1″), 3.38 (t, J = 9.0 Hz, H-2″), 3.35 (t, J = 9.0 Hz, H-3″), 3.43 (t, J = 9.0 Hz, H-4″), 3.96 (t, J = 9.5 Hz, H-5″). 13C NMR:d luteolin: 163.3 (C-2), 104.1 (C-2), 181.9 (C-4), 161.5 (C-5), 99.0 (C-6), 164.4 (C-7), 94.1 (C-8), 157.4 (C-9), 103.9 (C-10), 125.0 (C-1′), 113.9 (C-2′), 147.1 (C-3′), 148.2 (C-4′), 115.9 (C-5′), 118.6 (C-6′), glucuronide: 100.5 (C-1″), 73.0 (C-2″), 75.3 (C-3″), 71.5 (C-4″), 75.5 (C-5″), 170.3 (COOH)*.

### Ultraviolet visible (UV-vis), high resolution fast atom bombardment mass spectra (HR-FABMS), and nuclear magnetic resonance (NMR) measurements

UV-vis spectra for purified flavone was recorded on a UV-vis multi-purpose spectrophotometer (MPS-2450, Shimadzu Co. Ltd.) operated as a double-beam instrument in MeOH (from 200 to 500 nm). HR-FABMS were obtained in a positive ion mode using a 1:1 mixture of dithiothreitol and 3-nitrobenzyl alcohol as a matrix with the JMS-700 MS station mass spectrometer (JEOL Ltd., Japan). NMR spectra were recorded on a JNM AL-400 spectrometer (JEOL Ltd.) at 400 MHz for ^1^H spectra and 100 MHz for ^13^C spectra in dimethyl sulfoxide (DMSO)-*d*_6_. Chemical shifts are reported relative to DMSO (2.5 ppm) and coupling constants (*J*) are in Hz.

### Genetic analysis

The extraction of total RNA was performed by the RNeasy Plant Mini Kit (QIAGEN, Germany) and subjected to reverse transcription (RT) using an oligo-(dT)_20_ primer and ReverTra Ace (TOYOBO, Japan) according to the manufacture's instruction. RNA concentrations were adjusted to 100 ng/μl by spectrophotometer (Nanodrop, Thermo Scientific, USA). In “Concord,” the expression analysis was performed using RNA extracted from petals and leaves of white and blue monochromatic color plants (Figures 1E,F). In “Monique,” the central part (the colored part of the petal) and the peripheral part (the non-colored part) were cut with a razor and RNA was extracted from each area. For full length *flavanone 3-hydroxylase* (*SiF3H*; LC269962) expression in “Monique,” the petals and leaves of monochromatic plants (Figures 1H,I) were used for RNA extraction.

For the expressions of *SiCHS-A* (DQ788862): *chalcone synthase-A, SiF3H* (LC269962), *SiDFR* (LC269960): *dihydroflavonol 4-reductase, SiANS* (LC269961): *anthocyanidin synthase*, as genes coding enzymes on the anthocyanin biosynthesis pathway and *SiDEL* (LC269959): *DELILA*/*JAF13* (*a basic helix-loop-helix*), *SiMyb2* (LC269957), *SiWDR1* (LC269958), and *SiPAC* (LC269956) as transcriptional factors for anthocyanin biosynthetic genes were amplified by specific primers (Table S1) which were designed from their coding sequences. *SiActin* (AB596843) was used as the reference gene.

For DNA extraction, the DNeasy Plant Mini Kit (QIAGEN) was used according to the manufactures' instructions. Moreover, in order to extract DNA from the L1 layer, trichomes were collected by a razor blade under a stereomicroscope and the DNA was extracted by mixing with 2 ml of buffer AP1 (QIAGEN) in plastic tubes and extracted according to the manufactures' instructions. Also, the epidermis-removed petiole was used as a sample in order to obtain DNA from internal layers. DNA concentrations were adjusted below 2 ng/μl by spectrophotometer (Nanodrop, Thermo Scientific). All PCR/RT-PCR mixtures contained 1 μl of template DNA (at a maximum 1–2 ng), 0.1 μl of Blend *taq* (TOYOBO), and 0.2 μM of forward and reverse primers at a final volume of 10 μl according to the manufacture's instruction. The program was set at 94°C for 2 min, followed by 35 cycles at 94°C for 30 s, 55–60°C for 30 s depending on the value of the melting temperature of each primer set, and 72°C for 10–60 s depending on the length to be amplified. All PCR products were electrophoresed in 1% agarose gel and signals were detected under a UV-illuminator.

The 5′ flanking region sequence of *SiWDR1* in blue and white “Concord” mutants was determined by a commercial kit (RightWalk kit FK, BEX, Japan) and inverse PCR. According to the manufactures' instructions, genomic DNA (total 1 μg) of “Concord” leaves of monochromatic blue flowers was digested by *Xba* I (TaKaRa Bio, Japan) and ligated with adaptors supplied by the kit. The first PCR was conducted with WDR40 Walk R1st primer (Table S1) with WP-1 (supplied by the kit) and the second PCR was conducted by WDR40 Walk R2nd (Table S1) and WP-2 (supplied by the kit). The amplified product was cloned into pTAC-1 vector (BioDynamics Laboratory Inc., Japan) and sequenced by a 3,100 genetic analyzer (ABI PRISM, USA) after the reaction with a BigDye terminator v3.1 cycle sequencing kit (ABI PRISM). Genomic DNA (total 1 μg) of “Concord” blue and white mutants were digested with *Eco*RI (TaKaRa Bio) and self-ligated with 80 units of T4 DNA ligase (NEW ENGLAND BioLabs, USA). The ligated DNA was precipitated with ammonium acetate and ethanol and used for PCR. For the first PCR, 1st Primer-F aroundA and 1st Primer-R aroundA were used and for the nested PCR, B-F and 2nd Primer-R aroundA were used (Table S1). The product was cloned and sequenced as mentioned before. From the DNA sequence data of monochromatic blue and white flower plants, we could design primers for the determination of blue or white mutants, BF and BR primers for the blue mutant, and B-F and WDR white specific-R primers for the white mutant (Table S1).

To determine full length mRNA sequences of *SiF3H* for “Monique,” the GeneRacer™ Kit (Invitrogen, USA) was used according to the manufactures' instructions and a 5′-Full RACE Core set (TaKaRa Bio). The amplified products were cloned and sequenced as mentioned before. From the sequence data (LC2269962), primers for the total length amplification were designed and used for RT-PCR and genomic PCR using RNA/DNA of monochromatic blue flower plants. From these sequence data, the exon and intron structure of DNA of monochromatic blue flower plants was determined.

## Results

### Flower color separations in the regenerants of two *Saintpaulia* cultivars

Flowers of adventitious shoots from leaf laminas of “Concord” and “Monique” exhibited three types of flowers. In “Concord” white monochromatic-colored plants (L1 phenotype) (Figure 1E) were observed at the highest frequency (75.6%) followed by blue monochromatic-colored plants (L2 phenotype) (20.7%) (Table 2, Figure 1F). In “Monique” white monochromatic-colored plants (L1 phenotype) (Figure 1H) were observed at the highest frequency (45%), and 25% of regenerates were blue monochromatic plants (L2 phenotype) (Table 2, Figure 1I). Notably, three of the 82 “Concord” plants (3.7%) and six of the 20 “Monique” plants (30%) exhibited the same striped pattern as their mother plant (Table 2). On the other hand, the striped patterned flower mutant opposed to mother plants was not observed. When epidermis-peeled petioles of striped mother plants were used as explants, all flower color patterns in adventitious shoots were monochromatic and their color was identical to the center portion of the mother plant (Table 2). This result indicated that in both cultivars, inner cells did not pose genetic elements that produce white petals, and thus edged colors in mother plants had their origins in epidermal cells. Moreover, when monochromatic-colored regenerants from three cultivars were again used as explants for adventitious shoot regeneration, flower traits that developed in second regenerants retained the same flower traits as their first regenerants (Table 3). This result indicated that monochromatic-colored characteristics were stable during the adventitious shoot regeneration process, and two types of monochromatic-colored regenerants from “Concord” and “Monique” explants were genetically distinct from each other.

**Table 2:** Phenotypes of regenerated plants from different tissues of two cultivars.

**Cultivars**	**Explants**	**Number of plants (%)**			
		**No. of plants**	**L1 phenotype^b^**	**L2 phenotype^c^**	**L1 + L2 phenotype^d^**
Concord	Leaf disc	82	62 (75.6)	17 (20.7)	3 (3.7)
	Inner tissue^a^	20	0 (0)	20 (100)	0 (0)
Monique	Leaf disc	20	9 (45)	5 (25)	6 (30)
	Inner tissue	23	0 (0)	23 (100)	0 (0)

a *epidermis-peeled petiole was used*.

b *the monochromatic white flower, which is the same as the petal edge color*.

c *the monochromatic blue flower, which is the same as the petal edge centor*.

d *the pinwheel phenotype same as mother plants*.

**Table 3:** Phenotypes of regenerated plants from leaf discs of each monochormatic flower mutant of two cultivars.

**Cultivars**	**Flower color mutant**	**Number of plants (%)**			
		**No. of plants**	**L1 phenotype^a^**	**L2 phenotype^b^**	**L1 + L2 phenotype^c^**
Concord	Blue	80	0 (0)	80 (100)	0 (0)
	White	30	30 (100)	0 (0)	0 (0)
Monique	Blue	70	0 (0)	70 (100)	0 (0)
	White	80	80 (100)	0 (0)	0 (0)

a *the monochromatic white flower, which is the same as the petal edge color*.

b *the monochromatic blue flower, which is the same as the petal edge centor*.

c *the pinwheel phenotype same as mother plants*.

### Determination of anthocyanins in petals

Seven and nine anthocyanin derivatives were detected from “Concord” and “Monique” petals, respectively (Table 1) by HPLC analysis. The anthocyanidin moiety, malvidin, was revealed to be the main anthocyanidin in both cultivars (Table 1). Also, luteolin 4′-glucronide and apigenin 4′-glucronide were the main flavones for both cultivars (Table 1). The anthocyanin and flavone compositions in different mutants were not different (Table 1).

### Determination of candidate genes responsible for pigmentation variations in “Concord”

**Figure 2:**
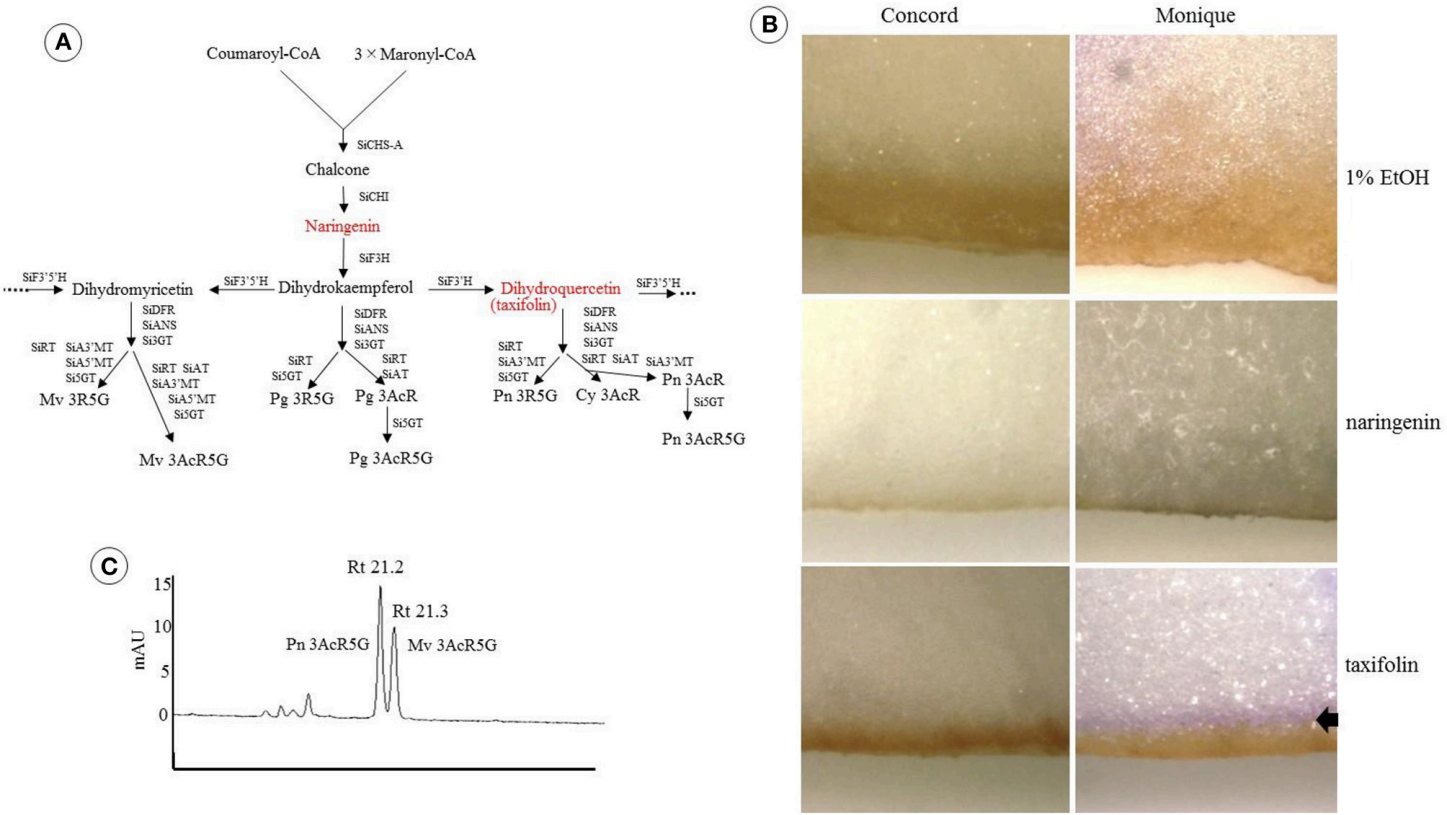
Feeding test of two precursors to *Saintpaulia* petals. **(A)** The positions of the two precursors (red) on the anthocyanin synthesis pathway and the final products are shown in Table 1. SiCHS-A, chalcone synthase-A; SiCHI, chalcone isomerase; SiF3H, flavone 3-hydroxylase; SiF3′ 5′H, flavonoid 3′ 5′-hydroxylase; SiF3′H, flavonoid 3′-hydroxylase; SiDFR, dihydroflavonol 4-reductase; SiANS, anthocyanidin synthase; Si3GT, 3-glucosyltransferase; SiRT, rhamnosyltransferase; SiAT, acyltransferase; Si5GT, 5-glucosyltransferase; SiA3′MT, anthocyanin 3′-methyltransferase; SiA5′MT, anthocyanin 5′-methyltransferase; Pg 3AcR, pelargonidin 3-acyl-rutinoside; Pg 3AcR5G, pelargonidin 3-acyl-rutinoside-5-glucoside; Pg 3R5G, pelargonidin 3-rutinoside-5-glucoside; Mv 3AcR5G, malvidin 3-acyl-rutinoside-5-glucoside; Mv 3R5G, malvidin 3-rutinoside-5-glucoside; Cy 3AcR, cyanidin 3-acyl-rutinoside; Pn 3AcR, peonidin 3-acyl-rutinoside; Pn 3AcR5G, peonidin 3-acyl-rutinoside-5-glucoside; Pn 3R5G, peonidin 3-rutinoside-5-glucoside. **(B)** The results of the feeding experiments of two precursors to the petals of “Concord” and “Monique.” The left column is the result for “Concord” and the right column is “Monique.” In the upper row, the results of the control group (1% ethanol), naringenin, and taxifolin are shown. Petals were photographed after incubating at 25°C for 24 h in light conditions. Arrow represents the colored parts. It seems that the brown stained areas observed in all samples were not caused by precursors but by ethanol. **(C)** HPLC result of colored part of “Monique” by taxifolin feeding. The number above the peak shows the retention time and the names next to each peak are the substances estimated from the absorbance of each peak.

**Figure 3:**
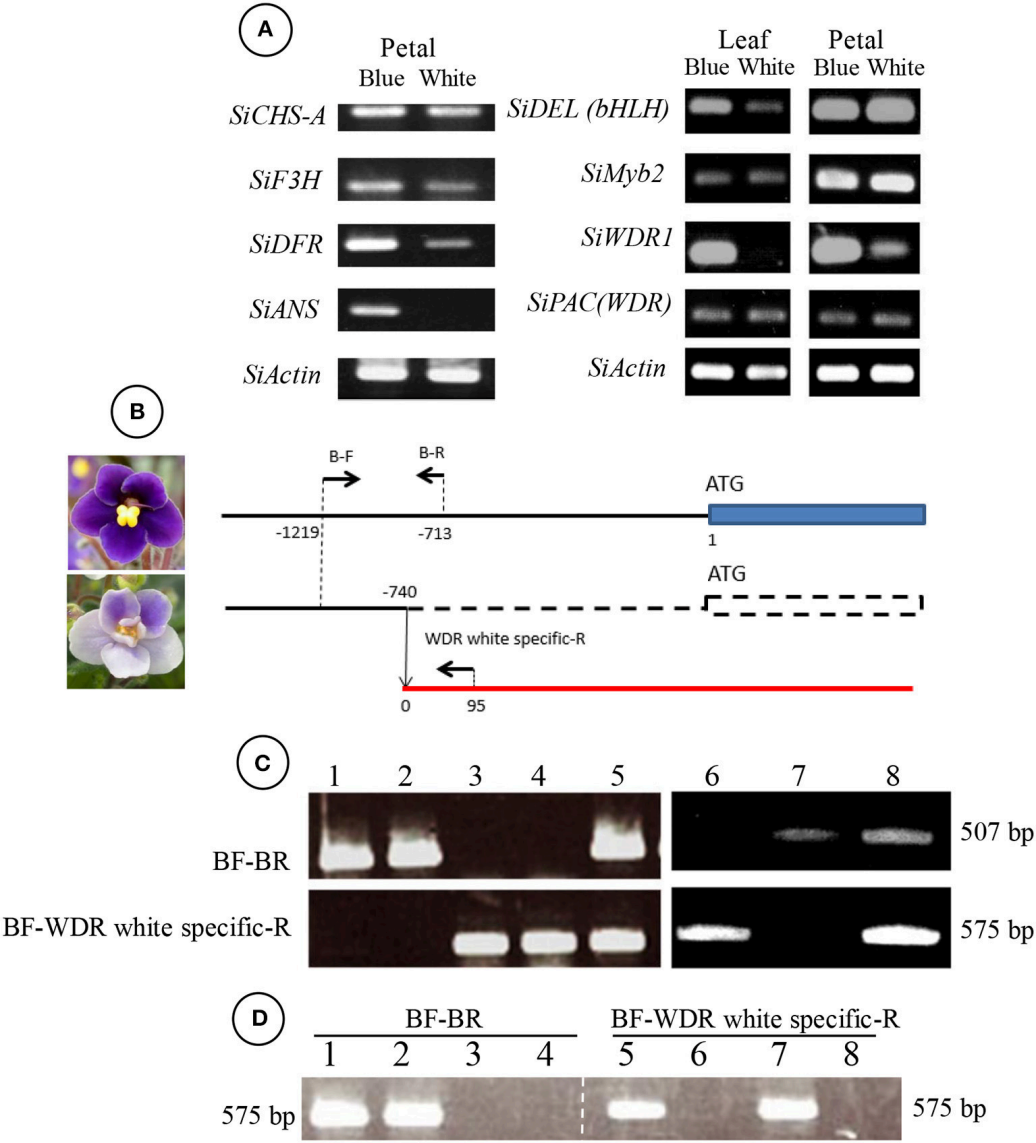
Genetic difference between blue and white mutants regenerated from “Concord” mother plants. **(A)** RT-PCR results of genes coding enzymes of the anthocyanin synthesis pathway (left panel) and transcriptional factors (right panel). The left panel indicates the results from blue and white flower petal mutants. The right panel indicates those of the leaf and petal. *SiCHS-A, chalcone synthase-A; SiF3H, flavanone 3-hydroxylase; SiDFR, dihydroflavonol 4-reductase; SiANS, anthocyanidin synthase; SiDEL(bHLH), DELILA/JAF13* (*a basic helix-loop-helix*). **(B)** Diagram of *SiWDR1* DNA of blue and white mutants. The arrows above the lines indicate the positions of primers used for **(C,D)**. *SiWDR1* DNA of the white mutant was found to be connected to an unknown gene. The numerical characters on the figure indicate the position of each primer that is from nucleotide “A” of the start codon ATG. **(C)** Genomic PCR results using two primer sets, BF-BR (upper panel) and BF-WDR white specific-R (lower panel). Leaves of blue flower mutants (1–2), white flower mutants (3–4), and a bi-color mother plant (5) were used. And, the trichome of leaves derived from L1 of the mother plant (6), inner cells of the petiole derived from L2 and L3 (7), and total leaf of the mother plant (8). **(D)** Genomic PCR results of SAMs of mother plants (1, 5), blue mutants (2, 6), and white mutants (3, 7). Lanes 4 and 8 are PCR products without templates (water).

**Figure 4:**
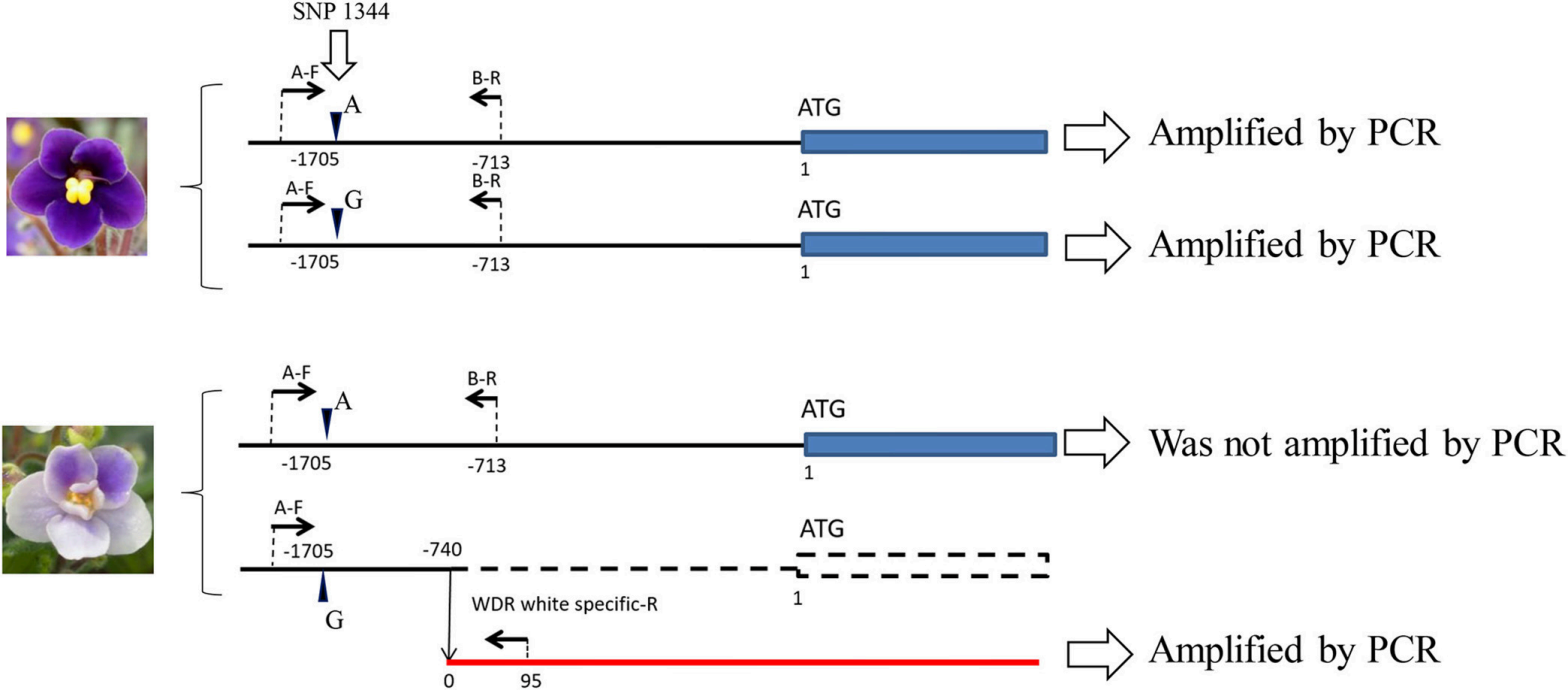
Two different types of *SiWDR1* gene detected in “Concord” mutants. In the blue mutant, two different types of *SiWDR1* were detected, which have a SNP difference at the 5′ flanking region. On the other hand, from white flower mutant, “A” type *SiWDR1*, was not detected. The rearrangement was observed in “G” type *SiWDR1*. Arrowheads indicate the SNP position and arrows indicate PCR primers.

When naringenin or taxifolin was fed to petals of white “Concord” mutants, pigment accumulation in the edge of the cut surface was not observed (Figure 2B), which indicated that steps after DFR either stopped or several steps in anthocyanin biosynthesis were stopped. The expressions of *SiCHS-A, SiF3H, SiDFR*, and *SiANS* in “Concord” petals were analyzed and the results showed that expressions of these genes, especially *SiF3H, SiDFR*, and *SiANS*, were lower in monochromatic white mutants compared to monochromatic blue mutants (Figure 3A). RT-PCR transcriptional factor results showed that *SiWDR1* expression in leaves was not detected in monochromatic white mutants and only a trace signal was detected monochromatic white petals (Figure 3A). Then, the upper stream of the *SiWDR1* DNA coding sequence was determined. In monochromatic blue mutants, SNPs upstream of the coding region indicated two types of *SiWDR1* sequences, hereafter called *SiWDR1-1* and *SiWDR1-2* (Figure 4). In monochromatic white mutants, we could not detect targeted *SiWDR1-1* and *SiWDR1-2* sequences when we used primers that amplified the 5′ flanking region. We also determined the sequence of the 5′ flanking region of monochromatic white mutants and revealed that the upstream region of *SiWDR1-2* was connected to an unknown sequence (Figure 3B). We designed a new primer, WDR white specific-R, which is located at the unknown sequence, and amplified the white specific sequence in combination with the B-F primer located at *WDR1* 5′ flanking region (Figure 3B). This combination successfully amplified the white genome but not blue genome (Figure 3C). On the other hand, a B-F and B-R combination amplified only the blue genome. The amplified sequences by A-F and B-R primers in blue mutants contains an SNP, A or G, which indicated *SiWDR1-1* and *SiWDR1-2*, respectively (Figure 4). The amplified sequence by A-F and WDR white specific-R contained the SNP “G,” which indicates the amplified sequence is *SiWDR1-2* (Figure 4). And trichome, which was originated from L1 of the mother plant, did not have the product by B-F and B-R primers that was in the mother plant (Figure 3). On the other hand, the inner cells that were derived from L2 or L3 of mother plants had a PCR product with B-F and B-R products, but did not have a product by B-F and WDR white specific-R (Figure 3C). The total leaves and meristems of mother plants have both products (Figures 3C,D). From these results, we could conclude that the mother plant has a periclinal chimeral structure that has L1 without functional SiWDR1 and inner layers with functional SiWDR1.

### Determination of candidate genes responsible for pigmentation variations in “Monique”

**Figure 5:**
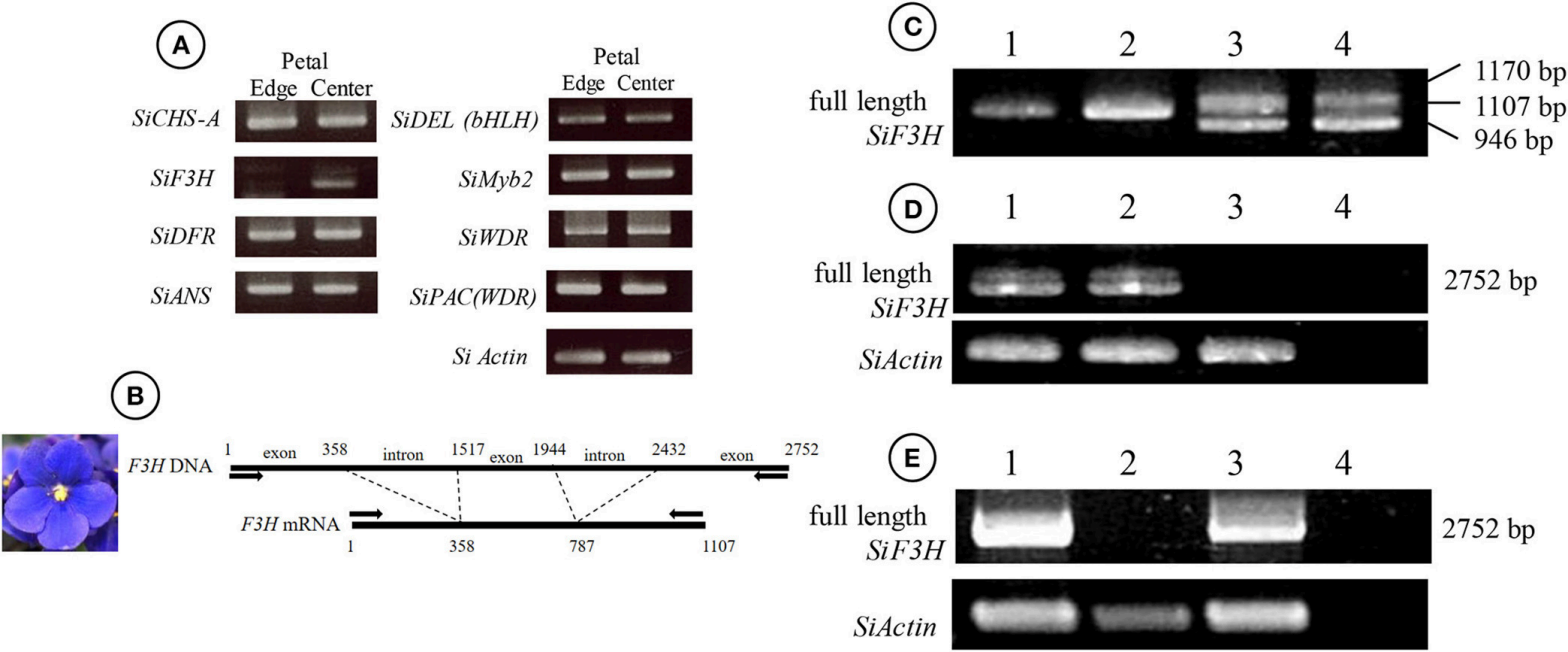
Genetic differences between blue and white mutants regenerated from mother plants of “Monique.” **(A)** RT-PCR results of genes coding enzymes of the anthocyanin synthesis pathway (left panel) and transcriptional factors (right panel). The left panel shows the petal results of blue and white flower mutants. The names of enzymes and transcription factors are referred to in Figure 3. **(B)** The diagram of *SiF3H* DNA (upper) and mRNA (lower) from the DNA of a blue flower mutant. The arrows on the lines indicate the primers used for **(C–E)**. The numerical characters on the figure indicate the position of splicing from nucleotide “A” of the start codon ATG. **(C)** RT-PCR results of full length of *SiF3H* mRNA. (1) Leaf of a blue flower mutant, (2) petal of a blue flower mutant, (3) leaf of a white flower mutant, (4) petal of a white flower mutant. On the lane of the blue flower mutant, a 1,107 bp band was observed and on the lane of the white flower mutant, 1,170 and 946 bp were observed. The lengths were determined by sequence analysis. **(D)** Genomic PCR for full length *SiF3H* DNA from leaves of the mother plant (1), blue flower mutant (2), white flower mutant (3), and control (water). **(E)** genomic PCR for full length *SiF3H* DNA from a total leaf of the mother plant (1), trichome from mother plants which were derived from L1 (2), inner cells of petioles derived from L2 and L3 (3), and control (water; 4). **(D,E)** primers for actin were used for actin DNA proliferation (lower panel).

Feeding naringenin to the petals of white edge “Monique” striped petals did not alter their pigmentation, but when fed taxifolin, the white petals turned reddish (Figure 2B). From these results, the candidate gene for the absence of malvidin derivatives in the white-colored phenotype was supposed to be an unfunction of F3H (Figure 2A). Anthocyanin from the portion fed taxifolin was revealed to be malvidin 3-acyl-rutinoside-5-glucoside, which is the main anthocyanin of this cultivar, and in this treatment, peonidin 3-acyl-rutinoside-5-glucoside was obtained (Figure 2C). The expressions of all anthocyanin biosynthetic genes except *SiF3H* were not different between monochromatic blue and white flower color mutants (Figure 5A). So, we determined the full length of *SiF3H* by 5′ and 3′ RACE. Using primers to amplify the full length of *SiF3H* mRNA (Table S1), one signal on the electrophoresed gel was detected in the monochromatic blue mutant, however, two bands that were different sizes compared to the blue one were detected. These two sequences have a 63 bp insertion (LC322156) and 161 bp deletion (LC322157) (Figure 5C, Figure S1), respectively. These two sequences in the monochromatic white mutant did not code complete protein sequences by stop codon generations (Figure S1). The genomic PCR results using full length primers in monochromatic white mutants In striped petals showed no *SiF3H* signal band (Figure 5D). The amplified product by the full length primers in the monochromatic blue mutant was sequenced and it was determined that the band was 2,752 bp. This sequence has two introns and three exons which completely coincided with the full length mRNA sequence of monochromatic blue mutants with 1,107 bp (Figure 5B). The DNA extracted from leaf trichomes of the mother plants were applied to genomic PCR of *SiF3H*, and the signal was not detected but was detected from inner tissues of petioles (Figure 5E). And, the full length *SiF3H* signal was detected in the mother plant. From these results, we can conclude that the mother plant has a periclinal chimeral structure which has L1 without functional SiF3H and inner layers with functional SiF3H.

### Observation of cell division at the formation of adventitious shoots

**Figure 6:**
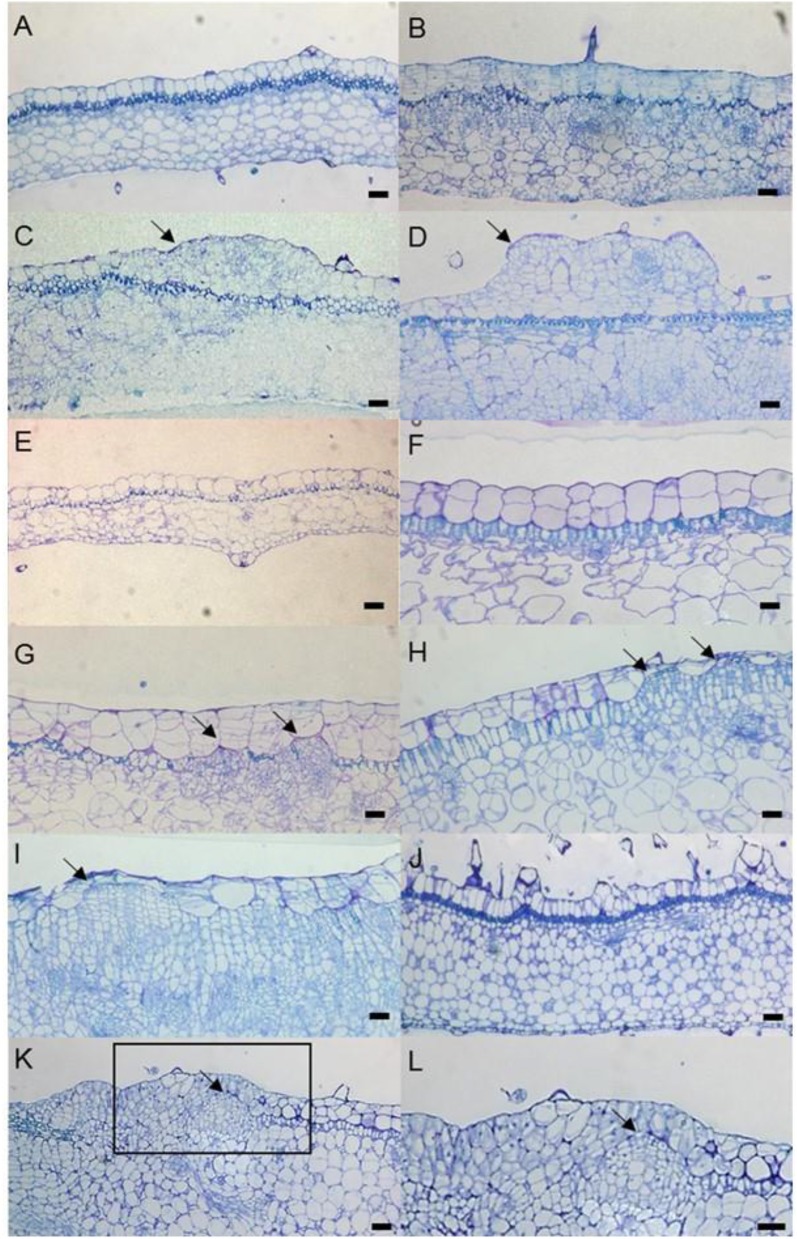
Time-course observation of cell division and SAM formation from leaf explants. “Kaname” leaf explants at 0 d **(A)**, 8 d **(B)**, 10 d **(C)**, and 14 d **(D)**. “Concord” leaf explants at 0 d **(E)**, 5 d **(F)**, 12 d **(G)**, and 18 d **(H,I)**. “Monique” leaf explants at 0 d **(J)** and 10 d **(K,L)**. **(L)** is a zoomed image of the rectangle in **(K)**. The arrows indicate the actively dividing cells. The arrows on **(C,D)** indicate cells derived from L1 and **(G,H)** indicate cells derived from L2. The scale bar = 100 μm.

**Figure 7:**
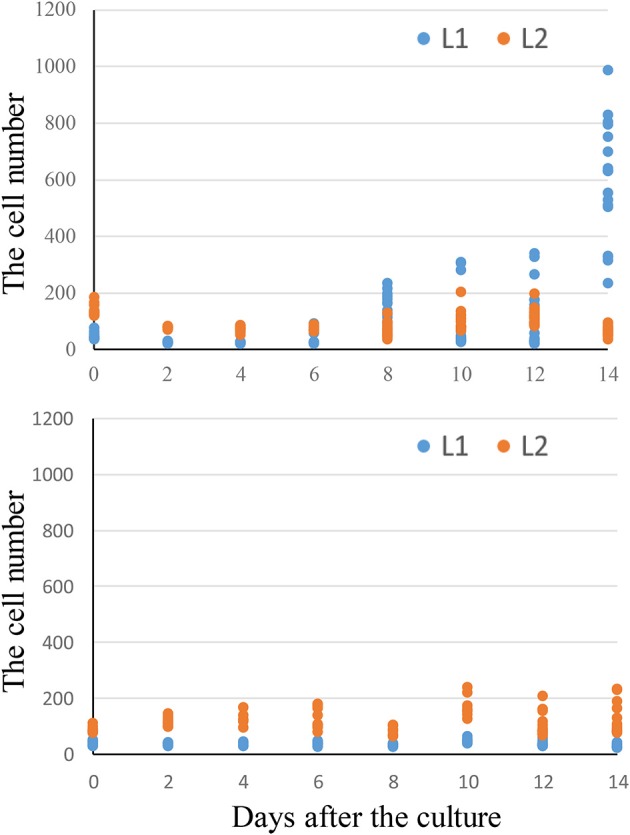
Time-course change in cell number from “Kaname” and “Concord” leaf disks. The y-axis indicates the total cell numbers in 2 mm length of leaf disks. The L1 cell number indicates the cells derived from adaxial epidermal cell divisions and L2 indicates the cell number derived from subepidermal cell divisions.

To determine the origins of adventitious shoots, histological observations were made on explants of “Kaname,” “Concord,” and “Monique” mother plants. At the start of culture, differences in leaf structures were not observed among the three cultivars and we could observe one epidermal and one subepidermal layer at the adaxial surface (Figures 6A,E,J). In “Kaname,” epidermal cells divided actively in the adaxial side 8 and 10 d after culture (Figures 6B,C) and shoot formation occurred in the epidermal cells at 14 d after culture (Figure 6D). In “Concord” explants, first cell divisions were observed 5 d after culture (Figure 6F), and at 12 d after culture, cell division at the epidermis was not so active and active cell division was instead observed at the subepidermal layer (Figure 6G). After that, cell clusters that had originated from the subepidermal layer pushed up the epidermis in “Concord” at 18 d after the culture (Figures 6H,I). In “Monique,” active cell division was also observed at the subepidermal layer rather than the epidermis10 d after culture (Figures 6K,L). Then, the number of divided cells in true-to-type “Kaname” and “Concord” explants were examined. In “Kaname,” the number of divided cells in the epidermis was much higher than those in subepidermal cells at 14 d after culture (Figure 7). In “Concord,” the number of cells in the epidermis did not increase during 14 d of culture. Instead, the number in subepidermal cells was a little higher than those in epidermal cells (Figure 7).

### The growth of plants regenerated from the epidermis and subepidermis

**Figure 8:**
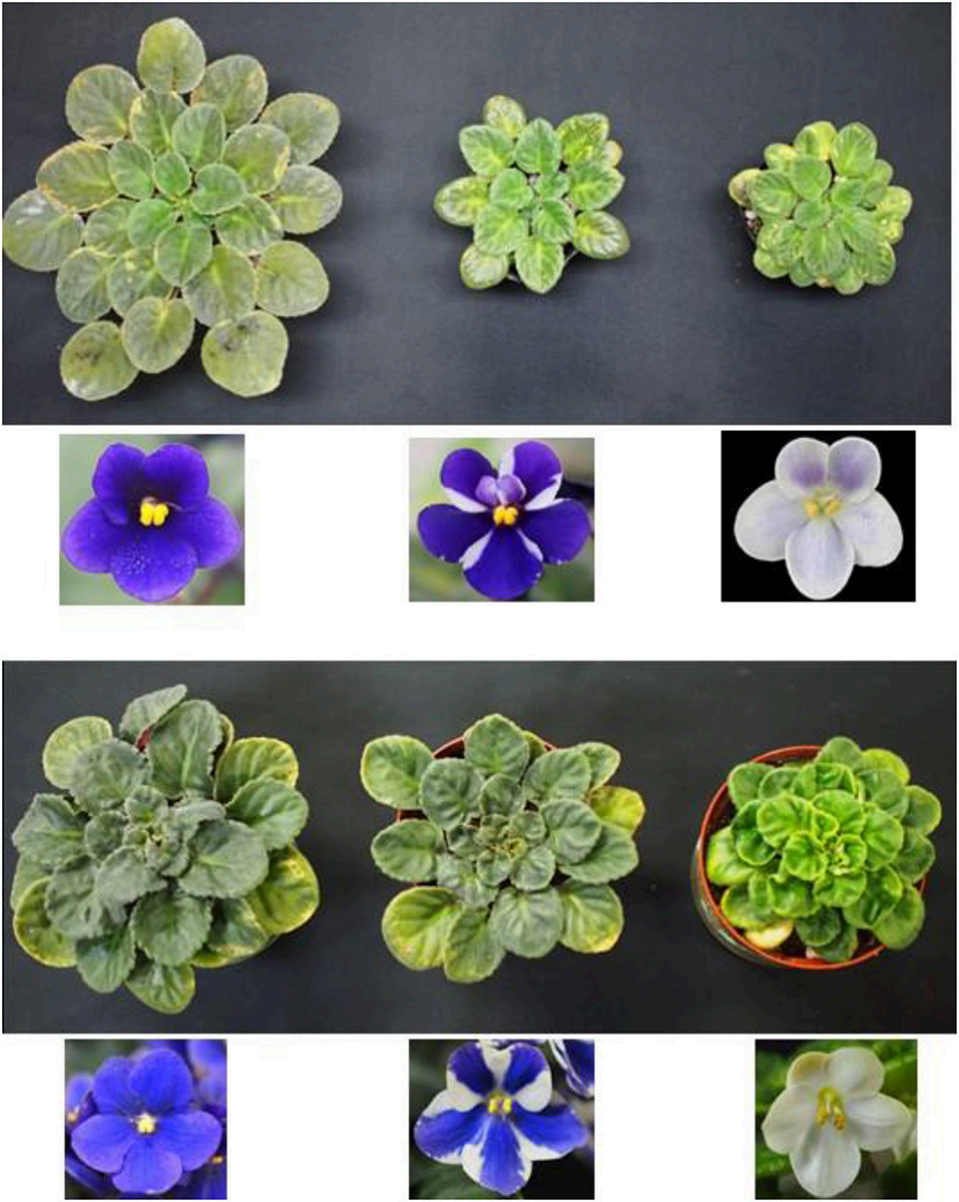
Growth differences of two cultivars. Blue flower color mutant (left), mother plant (middle), and white flower mutant (right) of “Concord” (upper) and “Monique” (lower). The photographs were taken with three plants lined side-by-side.

In “Kaname,” growth differences were not observed between monochromatic pink and blue mutants (Table 4, Figures 1B,C). In “Concord” and “Monique,” obvious growth differences were observed between the three types of plants; plant diameter and leaf sizes were smaller in epidermal-derived plants (monochromatic white) than inner layer-derived plants (monochromatic blue), and the mother plants had middle characteristics of these epidermis and inner-tissue originated plants (Table 4, Figure 8).

**Table 4:** The plant growth characteristics of mutants derived from each histogen layer of three cultivars.

**Cultivars**	**Type**	**Plant diameter (cm)**		**Largest leaf width (cm)**		**Largest leaf length (cm)**	
Kaname	Original	–		–		–	
	Blue	28.5	a	5.0	a	5.7	a
	Pink	27.1	a	4.9	a	5.6	a
Concord	Original	14.7	b	3.2	b	3.8	b
	Blue	23.1	a	5.1	a	5.2	a
	White	14.7	b	2.8	b	3.2	b
Monique	Original	14.1	b	4.1	a	4.4	a
	Blue	16.3	a	4.4	a	5.1	a
	White	11.6	c	3.4	a	3.3	b

*Data of original plant of “Kaname” were not collected because we could not propagate original plants*.

*The different alphabetical characters indicate the statistical differences between each cultivar*.

*For “Kaname” student t (p < 0.05) was used for 4 plants on each phenotype*.

*For “Concord” and “Monique,” Tukey-Kramer test (p < 0.05) was used for 6 and 4 plants, respectively*.

## Discussion

### “Kaname,” “Concord,” and “Monique” are periclinal chimeras

In *Saintpaulia*, there are bi-color cultivars, which have two different petal colors in the central and peripheral parts. This color-type cultivar is called pinwheel. When such cultivars are propagated by tissue culture, regenerated plants are separated into monochromatic flower color individuals (Lineberger and Druckenbrod, 1985; Ando et al., 1986; Peary et al., 1988; Sandall and Lineberger, 1997; Nielsen et al., 2003). However, in some cases the propagated plants maintain the pinwheel patterns. We should consider two causes for this result. One is because the striped pattern expression of some cultivars is caused by mechanisms other than periclinal chimera separation, e.g., by epigenetic instability. The other cause is because shoot regeneration is multi-celled in origin. Previous studies determined whether the patterned plants were periclinal chimeras or not based on their appearance, and estimated the origin of adventitious shoots also from the appearance of regenerated individuals. However, if a stripe pattern is formed by a mechanism other than a periclinal chimera, the origin of the adventitious shoots cannot be specified. Therefore, we first needed to obtain evidence that the specific origin of the mother plant was a periclinal chimera with a genetic mutation in the histogen layer.

From the HPLC result of “Concord,” the monochromatic white mutant, the anthocyanin and flavones greatly decreased but their composition did not be changed (Table 1). Feeding taxifolin did not induce anthocyanin biosynthesis in “Concord” petals, meaning that after DFR step it was at least un-functioned (Figures 2A,B). According to the reports on anthocyanin biosynthesis, a multistep inexpression of genes was considered to be attributed to transcription factors (Koes et al., 2005). The decreased expressions of the four genes analyzed indicated that a transcriptional factor, WDR1, was supposed to be the strong candidate for the white phenotype (Figure 3A). The sequence of the trace band observed in white petals (Figure 3A) had nine SNP differences from the amplified *SiWDR1-1* and *SiWDR1-2* sequences using same primers (data not shown), and this product was not petal-specific because the direct sequence of RT-PCR product contained the nine SNPs sequences in addition to the *SiWDR1* sequence (data not shown). Suppression of these enzymatic genes, including *SiANS* which is after the *SiDFR* step, was consistent with the taxifolin feeding experimental result. SiWDR1 plays the most important role for anthocyanin biosynthesis by complexing with other transcriptional factors and by regulating the expression of several enzymatic genes on the anthocyanin biosynthesis pathway (Koes et al., 2005). In “Concord,” two *SiWDR1* genes were determined in blue-type cells, one of which, the 5′ flanking region of *SiWDR* (*SiWDR1-2*), was connected to an unknown sequence (Figure 3B). This unknown sequence was also in blue mutant cells, but the 5′ flanking region of this unknown sequence was quite different from the white mutant cells (data not shown). This indicates that the white mutant *SiWDR1-2* lost its function by spontaneous genetic rearrangement with other DNA sequence. Further, another *SiWDR* (*SiWDR1-1*) had disappeared from white mutant cells (Figure 4). We cannot discuss why the *SiWDR1-1* disappeared from the genome, but one hypothesis is that these two *SiWDR1* were located at a same locus and the spontaneous rearrangement of *SiWDR1-2* and the disappearance of *SiWDR1-1*occurred simultaneously.

In “Monique,” HPLC results revealed that this cultivar also decreased anthocyanin and flavone accumulation, but their composition was not changed (Table 1). This also indicated that upstream of the anthocyanin biosynthesis pathway is inactivated. Only feeding taxifolin to petals induced anthocyanin biosynthesis, which indicates that the steps after F3′5′H and DFR were normally activated and malvidin 3-acyl-rutinoside-5-glucoside (Table 1), the main anthocyanin of the blue area of “Monique” petals, were detected from the painted area by HPLC, which indicated that the step after SiF3′5′H was active (Figures 2A,C). Biosynthesis of peonidin 3-acyl-rutinoside-5-glucoside was observed in the taxifolin treatment, which indicates that SiF3′H was also active (Figure 2A). As expected in this cultivar, DNA sequence differences were observed in *SiF3H* (Figure 5). Monochromatic white mutant full length genomic *SiF3H* was not amplified by ordinal PCR (Figure 5), but the long PCR method was conducted using the same template and about an 8 kb band was observed. We did not get the sequence of the band, but this band evoked an inserted sequence such as a transposable element. From these evidences, we concluded that “Concord” and “Monique” were periclinal chimeras, the same as our previous study on “Kaname” which has a mutated SiF3′5′H in the L1 layer.

In addition to these genetic evidences, the results of repetition of shoot regeneration from the monochromatic-colored mutants, in which the monochromatic color expression was stable, supports the conclusion that the used cultivars were periclinal chimeras and allowed us to determine adventitious shoot origin.

### Does shoot formation occur from single or multi-cell layers in *Saintpaulia*?

We found that the origin of shoot formation was different depending on the cultivar. Many histological observations have revealed that shoot formation occur from the epidermis (Naylor and Johnson, 1937; Arisumi and Frazier, 1968; Broertjes and Keen, 1980; Ohki, 1994; Hosokawa et al., 1998). In *Saintpaulia*, epidermal cells are easier to divide and form new meristems. Yang et al. (2017) revealed that 100% of “Kaname” shoots were a monochromatic flower color and were the same as the epidermal color phenotype which was derived from the L1 layer in the mother plant. On the other hand, shoot formation of “Concord” and “Monique” were solely from L1 or L2 and sometimes from both L1 and L2 (Table 2). Therefore, it is certain that adventitious shoots are formed by multiple cells derived from multiple histogen layers in these cultivars.

**Figure 9:**
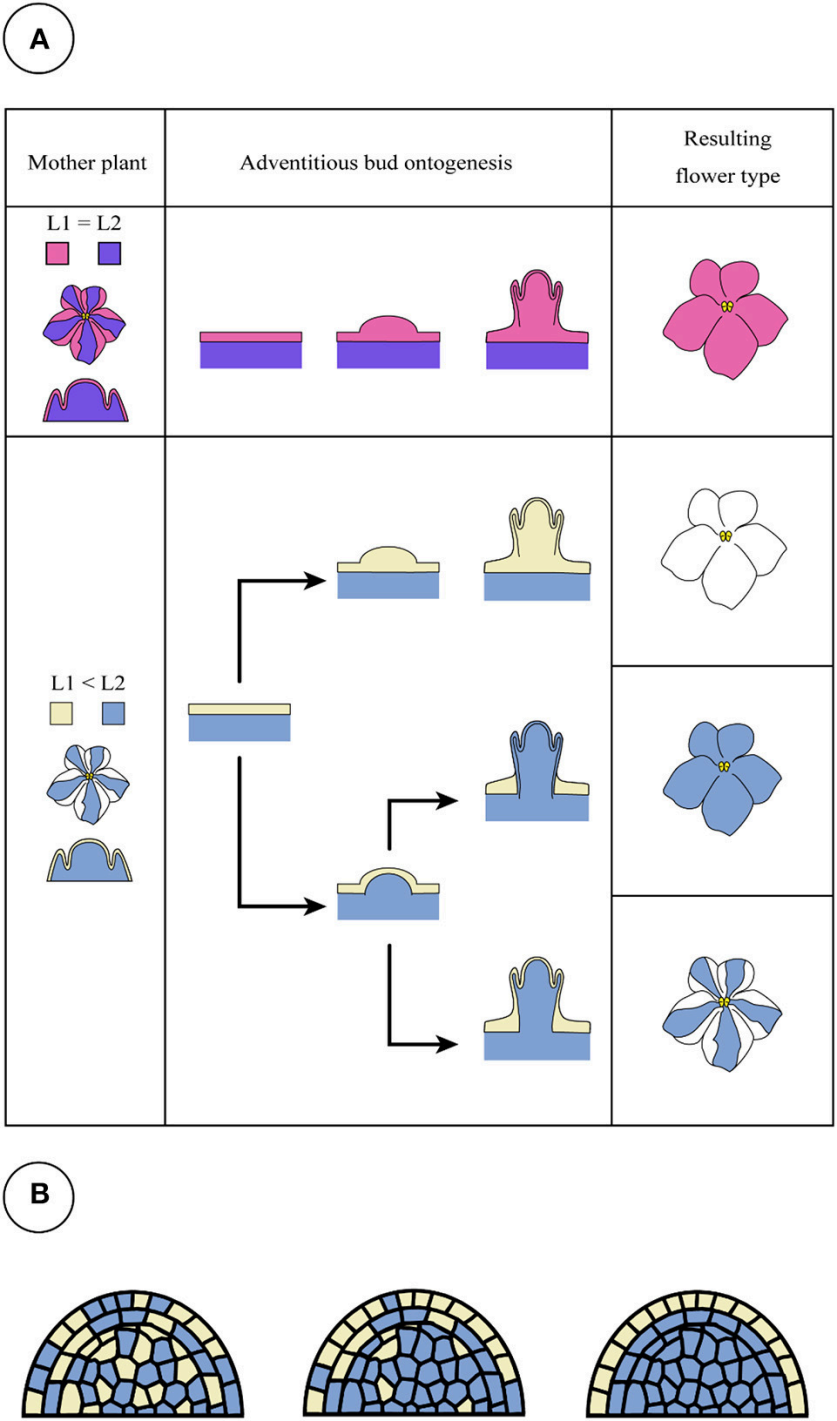
Candidate patterns for adventitious shoot formation of *Saintpaulia*. When the cell division activities of L1 and L2 are equal or L1 is higher than L2, a shoot is generated from epidermal cells derived from the L1 layer (**A**, upper panel). This pattern is found in most *Saintpaulia* cultivars. When epidermal cells derived from the L1 layer have low cell division activities, adventitious buds will be generated from the L2 cells (**A**, lower panel) in a state where the L1 layer is involved. In this case, three types of plants will be observed. Although all adventitious buds with a peripheral chimeral structure have white type cells in their L1 layer, two hypotheses were conceived for the occurrence pattern of the individuals. One is a pattern in which the L2 layer pushes up the L1 layer **(A)**, but is a mosaic. It is not possible to deny the possibility that **(B)** the shoot apical meristem generated as a mosaic with genetically different cells will develop into the stable structure (from left to right panel), white cells in L1 layer in this case.

However, a new question arises as to what determines the origin of shoot formation. In “Kaname,” all of the regenerated shoots from the leaves of mother plants developed monochromatic pink flowers which had a nonfunctional *SiF3*′*5*′*H* allele, indicating that all adventitious shoots were regenerated from the epidermis. This type of chimera separation has been frequently reported (Abu-Qaoud et al., 1990). The mutation of *SiF3*′*5*′*H* will not reduce their growth vigor (Table 4). Before cell division starts in subepidermal tissues, meristem formation occurred at the epidermal cells (Figure 9A). In contrast to this case, cell division of the epidermis in “Concord” and “Monique” was slow and rather it was observed that the subepidermal cells divided (Figures 6, 7). Regenerant “Concord” and “Monique” leaves developed monochromatic white, monochromatic blue, and striped flowers, and more than 20% of shoots of total shoots were derived from subepidermal tissues in both cultivars (Table 2). Histological observations in cultured explants supported this idea, and active cell division was observed in subepidermal cells in “Concord” and “Monique” during culture (Figure 6). A comparative histological analysis among “Kaname,” “Concord,” and “Monique” suggested that a relatively high frequency of cell division activities in subepidermal cells explained the occurrence of non-chimeral, inner layer type regenerants from leaf explants. In conclusion, when the cells with a weak vigor cover vigorous cell layers, inner cells start to divide before the epidermal cells and form new SAMs by pushing up the L1 layer (Figures 6, 9A). Sometimes, the inner cells burst L1 cells during the formation of SAMs, in this instance the genotypes of shoots were L2 blue flowered plants in “Concord” and “Monique.” In some cases, L2 cells formed SAMs while pushing up the L1 layer, in this instance the shoot genotype was chimeral, the same structure as the mother plant. In some cases, the L1 cell layer divided solely and formed SAMs, in this instance the shoot genotype is the L1 layer, the white flower plants in these cultivars. Shoot regeneration occurs from the most vigorous cell layers, L1 in *Saintpaulia*, but in cases where some mutations decrease cell vigor in the cell layer, adventitious shoots regenerate from other cell layers solely or include some cell layers (Figure 9A).

The third question is what kind of phenotype determined the cell division activities in those cultivars. In the “Kaname” mother plant, the structure of its SAM was considered to be composed by L1 carrying mutated, nonfunctional *SiF3*′*5*′*H* and inner layers carrying non-mutated, functional *SiF3*′*5*′*H*. All of the regenerated shoots from the leaves of mother plants developed monochromatic pink flowers which had a nonfunctional *F3*′*5*′*H* allele, indicating that all the adventitious shoots were regenerated from the epidermis. In addition, commercial striped *Saintpaulia* cultivars interestingly have petals with a blue and pink pattern. This color pattern is similar to “Kaname” and is due to their easy growing. The mutation of *SiF3*′*5*′*H* was not supposed to affect cell division activity. On the other hand, type such as “Concord” and “Monique” which have colorless layers are difficult to grow because their growth vigor is low (Table 4, Figure 8). Thus, this type of cultivars is very rare in horticultural cultivars. This slow vigor seems to due to differences in the stop position of anthocyanin biosynthetic genes. Mutations in genes on the anthocyanin synthesis pathway generally do not affect the growth of the plant (Holton and Cornish, 1995). However, in *Saintpaulia*, the mutation of anthocyanin biosynthesis genes, at least *SiWDR* or *SiF3H* mutations, will contribute to a low vigor of cell divisions.

### Why were all regenerated shoots with a periclinal chimeral structure true-to-type?

In *Saintpaulia*, the trait of anthocyanin biosynthesis under the L2 layer affects the coloring of the L1 layer (Matsuda et al., 2014), so the white-colored mutant of the L2 layer should appear as a pale stripe in the center of a petal. However, in “Concord” and “Monique,” we could not obtain L2-mutated periclinal chimeral plants. When a meristem is composed of different cells and types of cells have a different vigor, vigorous cells will dominate and the chimeral structure will disappear. In fact, we could find some mericlinal or sectorial chimera in “Kaname” but could not find any these types of chimeras in “Concord” and “Monique.” Sato et al. (2011) cultured the leaf lamina of *Saintpaulia* “Thamires” which has blue splotches on pink petals and obtained various mutants in the regenerants. The cause of the blue splotches is spontaneous transposition of the *h*AT superfamily transposoable element (TE) from the *SiF3*′*5*′*H* promoter, and the blue color was determined to be a malvidin derivative and the pink pigment a pelargonidin derivative. During shoot regeneration, TE was transposed from the promoter region and many monochromatic blue mutants and periclinal mutants were obtained. Some of the periclinal chimera was L1 pink and L2 blue, which is the same pattern as “Kaname,” and the others were L1 blue and L2 pink which is the opposite color pattern to “Kaname.” So, this means that a periclinal chimera structure with a mutated *SiF3*′*5*′*H* L2 is possible. If the vigorous L1 covered the non-vigorous inner layer, it would be difficult for this type of periclinal chimera to maintain its structure. We often find some leaf variegated plants whose L1 are an unvigorous albino genotype, and which have been maintained for many years in vegetative propagation. This suggests that when the vigorous L2 is covered by weak L1, it makes the chimeral meristem structure stable. We can suppose that L1 cells are much vigorous than L2 cells. L1 cells will invade into the L2 and break the chimera structure. So, if the cells with a different vigor compose a SAM, the low-vigor cells will distribute at the outer cell layer, L1.

Two possibilities can be considered in the process of shoot regeneration of a periclinal chimera. One is a case where the L2 layer develops SAMs that include cells of the L1 layer, and the other is a case where a mosaic SAM with L1 and L2 cells occur and more stable periclinal structured SAMs are screened (Figure 9B). Here, we can observe shoot regeneration from L2 cells pushing up L1 cells, however, the latter possibility cannot be abandoned based on the results from this study.

In conclusion, we propose that the striped *Saintpaulia* cultivars, whose patterns are derived from a periclinal chimera structure, can be divided into two types. One type is cultivars whose regenerated shoots are derived from the epidermal layer, such as “Kaname,” whose epidermis undergoes active cell division during tissue culturing. Another type is cultivars that have a relatively high percentage of regenerated adventitious shoots that strictly adhere to their mothers' original chimeral structures, such as “Concord” and “Monique,” whose inner cells but not epidermal cells undergo active cell division during tissue culturing. The latter type of cultivars may be recognized by comparing growth characteristics of separated epidermis-derived plants and inner-derived plants, which show vigorous and less vigorous growth, respectively.

## Author contributions

TN contributed to data collection, data analysis, and writing the draft manuscript; SY, SO, AD, and MD contributed to data analysis, data interpretation, drafting the work, and final approval of the version; KH and FT contributed to data collection, data analysis, data interpretation, drafting the work, and final approval of the version; MH contributed to the conception or design of the work, data analysis, data interpretation, writing the draft manuscript, and final approval of the version.

### Conflict of interest statement

The authors declare that the research was conducted in the absence of any commercial or financial relationships that could be construed as a potential conflict of interest.
