# Supplementary material for: Histogen Layers Contributing to Adventitious Bud Formation Are Determined by their Cell Division Activities
Source: Front Plant Sci. 2017 Oct 17;8:1749. doi: 10.3389/fpls.2017.01749 (PMC5650975; doi:10.3389/fpls.2017.01749)
Supplement: Supplementary file 1 [file DataSheet1.PDF]

>White type 946 bp (LC322156)
ATGGCAAAAACACTGACAGAACAAGCACAACAAGCATCCCTTCCCCCGAATTTCATCCGGGACGAAGACGAAAGGCCGAAGGT
CGCGTATAACGACTTTAGCGTCGACATTCCGGTGATATCCATCGCCGGGATCGACGAATCTGGCCACCGCAGGGAGGAAGTGTG
CCGGAAAATTGTCGCGGCTTGCGAAGACTGGGGGATTTTTCAGGTGATCGATCATGGGGTCGATGCGAAACTCATAACGGAAAT
GACTCGTTTGGCTCGTGAGTTTTTCGACTTGCCGCCGCTGGAGAAGCTCCGTTACGATATGAGCGGTGGCAAGAAAGGTGGTTTT
ATTGTTTCCAGCCATTTGCAGGGTGAAAGCGTGCAAGACTGGAGAGAAATTGTGACATATTTTTCGTACCCGATCGAGGCCCGG
GATTACTCGAGATGGCCCGAGAAGCCCGAGGGATGGCGGGCGGTGACGGAGGCCTATAGCGAGCAGATGATGAATCTTGCTTG
CAAATTGTTGGAGGTTTTATCAGAGGCAATGGGACTTGACAAGGATGCATTAACTAAAGCCTGTGTGGACATGGACCAAAAGGT
TGTGGTCAACTACTACCCAAAATGCCCTCAACCCGATCTCACACTCGGGCTCAAACGACACACGGATCCGGGTACGATCACTTT
GCTGCTCCAGGACCAGGTTGGCGGGTTGCAGGCGACCCGGGATGGCGGCCAAACATGGATCACGGTTCAGCCTGTCGAAGGGG
CTTTTGTTGTCAATCTTGGTGACCATGGTCATTGCTGTCACTGGGACCAACAAGAGGAAGATGGCCAATGATCTGGAGCGTGCG
AGGCTCAAGAAATTGGCTAAGGAACTAGCTGAGGTTGAGGTGAAGAGCAATACCAAGATTCCTGTTAATGTTAAAACATCCAA
AGGCATAGAAGAGATTCTTGCTTGA

>White type 1170 bp ((LC322157)
ATGGCAAAAACACTGACAGAACAAGCACAACAAGCATCCCTTCCCCCGAATTTCATCCGGGACGAAGACGAAAGGCCGAAGGT
CGCGTATAACGACTTTAGCGTCGACATTCCGGTGATATCCATCGCCGGGATCGACGAATCTGGCCACCGCAGGGAGGAAGTGTG
CCGGAAAATTGTCGCGGCTTGCGAAGACTGGGGGATTTTTCAGGTGATCGATCATGGGGTCGATGCGAAACTCATAACGGAAAT
GACTCGTTTGGCTCGTGAGTTTTTCGACTTGCCGCCGCTGGAGAAGCTCCGTTACGATATGAGCGGTGGCAAGAAAGGTGGTTTT
ATTGTTTCCAGCCATTTGCAGGGTGAAAGCGTGCAAGACTGGAGAGAAATTGTGACATATTTTTCGTACCCGATCGAGGCCCGG
GATTACTCGAGATGGCCCGAGAAGCCCGAGGGATGGCGGGCGGTGACGGAGGCCTATAGCGAGCAGATGATGAATCTTGCTTG
CAAATTGTTGGAGGTTTTATCAGAGGCAATGGGACTTGACAAGGATGCATTAACTAAAGCCTGTGTGGACATGGACCAAAAGGT
TGTGGTCAACTACTACCCAAAATGCCCTCAACCCGATCTCACACTCGGGCTCAAACGACACACGGATCCGGGTACGATCACTTT
GCTGCTCCAGGACCAGGTTGGCGGGTTGCAGGCGACCCGGGATGGCGGCCAAACATGGATCACGGTTCAGCCTGTCGAAGGGG
CTTTTGTTGTCAATCTTGGTGACCATGGTCATTATCTAAGCAATGGGAGATTCAAGAATGCTGATCACCAAGCAGTAGTGAACTC
AAACAGTAGCAGACTATCGATAGCAACATTCCAGAATCCAGCACTGGATGCCACTGTTTACCCTTTAAAGATCAGAGACGGGGA
CAAACCAATACTCGACGAACCGATAACGTTTTCGGAGATGTACAAGAGGTGTCAGAAACCAGAAACCAATTAGTCAACAAAAT
ACAGTGCTGTCACTGGGACCAACAAGAGGAAGATGGCCAATGATCTGGAGCGTGCGAGGCTCAAGAAATTGGCTAAGGAACTA
GCTGAGGTTGAGGTGAAGAGCAATACCAAGATTCCTGTTAATGTTAAAACATCCAAAGGCATAGAAGAGATTCTTGCTTGA

>Blue type 1107 bp (LC269962)
ATGGCAAAAACACTGACAGAACAAGCACAACAAGCATCCCTTCCCCCGAATTTCATCCGGGACGAAGACGAAAGGCCGAAGGT
CGCGTATAACGACTTTAGCGTCGACATTCCGGTGATATCCATCGCCGGGATCGACGAATCTGGCCACCGCAGGGAGGAAGTGTG
CCGGAAAATTGTCGCGGCTTGCGAAGACTGGGGGATTTTTCAGGTGATCGATCATGGGGTCGATGCGAAACTCATAACGGAAAT
GACTCGTTTGGCTCGTGAGTTTTTCGACTTGCCGCCGCTGGAGAAGCTCCGTTACGATATGAGCGGTGGCAAGAAAGGTGGTTTT
ATTGTTTCCAGCCATTTGCAGGGTGAAAGCGTGCAAGACTGGAGAGAAATTGTGACATATTTTTCGTACCCGATCGAGGCCCGG
GATTACTCGAGATGGCCCGAGAAGCCCGAGGGATGGCGGGCGGTGACGGAGGCCTATAGCGAGCAGATGATGAATCTTGCTTG
CAAATTGTTGGAGGTTTTATCAGAGGCAATGGGACTTGACAAGGATGCATTAACTAAAGCCTGTGTGGACATGGACCAAAAGGT
TGTGGTCAACTACTACCCAAAATGCCCTCAACCCGATCTCACACTCGGGCTCAAACGACACACGGATCCGGGTACGATCACTTT
GCTGCTCCAGGACCAGGTTGGCGGGTTGCAGGCGACCCGGGATGGCGGCCAAACATGGATCACGGTTCAGCCTGTCGAAGGGG
CTTTTGTTGTCAATCTTGGTGACCATGGTCATTATCTGAGCAATGGGAGATTCAAGAATGCTGATCACCAAGCAGTGGTGAACTC
AAACAGTAGCAGACTATCGATAGCAACATTCCAGAATCCAGCACTGGATGCCACTGTTTACCCTTTAAAGATCAGAGACGGGGA
CAAACCAATACTCGACGAACCAATAACTTTTTCCGAGATGTACAAGAGGAAGATGGGCAATGATCTGGAGCGTGCGAGGCTCA
AGAAATTGGCTAAGGAACTAGCTGAGGTTGAGGTGAAGAGCAATACCAAGCTTCCTGTTAATGTTAAAACATCCAAAGGCATA
GAAGAGATTCTTGCTTGA

White type 946 bp (LC322156)
MAKTLTEQAQQASLPPNFIRDEDERPKVAYNDFSVDIPVISIAGIDESGHRREEVCRKIVAACEDWGI
FQVIDHGVDAKLITEMTRLAREFFDLPPLEKLRYDMSGGKKGGFIVSSHLQGESVQDWREIVTYFSY
PIEARDYSRWPEKPEGWRAVTEAYSEQMMNLACKLLEVLSEAMGLDKDALTKACVDMDQKVVVN
YYPKCPQPDLTLGLKRHTDPGTITLLLQDQVGGLQATRDGGQTWITVQPVEGAFVVNLGDHGHCC
HWDQQEEDGQ-SGACEAQEIG-GTS-G-GEEQYQDSC-C-NIQRHRRDSCL

White type 1170 bp (LC322157)
MAKTLTEQAQQASLPPNFIRDEDERPKVAYNDFSVDIPVISIAGIDESGHRREEVCRKIVAACEDWGI
FQVIDHGVDAKLITEMTRLAREFFDLPPLEKLRYDMSGGKKGGFIVSSHLQGESVQDWREIVTYFSY
PIEARDYSRWPEKPEGWRAVTEAYSEQMMNLACKLLEVLSEAMGLDKDALTKACVDMDQKVVVN
YYPKCPQPDLTLGLKRHTDPGTITLLLQDQVGGLQATRDGGQTWITVQPVEGAFVVNLGDHGHYL
SNGRFKNADHQAVVNSNSSRLSIATFQNPALDATVYPLKIRDGDKPILDEPITFSEMYKRCQKPETN-
STKYSAVTGTNKRKMANDLERARLKKLAKELAEVEVKSNTKIPVNVKTSKGIEEILA-

Blue type 1107 bp (LC269962)
MAKTLTEQAQQASLPPNFIRDEDERPKVAYNDFSVDIPVISIAGIDESGHRREEVCRKIVAACEDWGI
FQVIDHGVDAKLITEMTRLAREFFDLPPLEKLRYDMSGGKKGGFIVSSHLQGESVQDWREIVTYFSY
PIEARDYSRWPEKPEGWRAVTEAYSEQMMNLACKLLEVLSEAMGLDKDALTKACVDMDQKVVVN
YYPKCPQPDLTLGLKRHTDPGTITLLLQDQVGGLQATRDGGQTWITVQPVEGAFVVNLGDHGHYL
SNGRFKNADHQAVVNSNSSRLSIATFQNPALDATVYPLKIRDGDKPILDEPITFSEMYKRKMGNDLE
RARLKKLAKELAEVEVKSNTKLPVNVKTSKGIEEILA-

Fig. S1. Three different nucleotide and deduced amino acid sequences of SiF3H of “Monique”.
White 946 bp and 1170 bp were detected from a white flower mutant and blue 1107 bp was detected from a blue flower mutant. The arrows indicate the stop codon and this suggests that the white color type does not code complete protein. The red colored characters are protein sequences.
