# Supplementary material for: Histogen Layers Contributing to Adventitious Bud Formation Are Determined by their Cell Division Activities
Source: Front Plant Sci. 2017 Oct 17;8:1749. doi: 10.3389/fpls.2017.01749 (PMC5650975; doi:10.3389/fpls.2017.01749)
Supplement: Supplementary file 2 [file Table1.PDF]

Table S1. Primers used for the experiment.

Primer name	Target	Methods	Sequences (5'-3')	Result	Accession numbe
SiCHSA-F	*chalcone synthase-A*	RT-PCR	gcgagactcatctggatagctt	Fig. 2	DQ788862
SiCHSA-R	*chalcone synthase-A*	RT-PCR	gcttccctcaaacttttctcaa	Fig. 2	DQ788862
SiDFR-F	*dihydroflavonol 4-reductase*	RT-PCR	tgtgagggcgtgttccacat	Fig. 2	LC269960
SiDFR-R	*dihydroflavonol 4-reductase*	RT-PCR	ccatcctgtcattttagtggag	Fig. 2	LC269960
SiANS-F	*anthocyanidin synthase*	RT-PCR	atgctagtgggaagttggagtg	Fig. 2	LC269961
SiANS-R	*anthocyanidin synthase*	RT-PCR	gggcatatcgggtaatagttga	Fig. 2	LC269961
SiF3H-F	*flavanone 3-hydroxylase*	RT-PCR	atcactttgctgctccaggac	Fig. 2	LC269962
SiF3H-R	*flavanone 3-hydroxylase*	RT-PCR	ttgaacctcccattgctta	Fig. 2	LC269962
SiMyb2-F	*Myb2*	RT-PCR	accatcggtgtgaagaaagg	Fig. 2	LC269957
SiMyb2-R	*Myb2*	RT-PCR	gtccggtgaggaaggtatga	Fig. 2	LC269957
SiDEL-F	*DELILA/JAF13 (a basic helix-loop-helix)*	RT-PCR	ctataatggcgatatcaagacc	Fig. 2	LC269959
SiDEL-R	*DELILA/JAF13 (a basic helix-loop-helix)*	RT-PCR	gagagaaaactttggtgtctg	Fig. 2	LC269959
SiWDR-F	*WDR*	RT-PCR	taccatctgggatatcgaaagg	Fig. 2	LC269958
SiWDR-R	*WDR*	RT-PCR	gccaacctcaacagaggtgtat	Fig. 2	LC269958
SiPAC-F	*PAC*	RT-PCR	tgtttgaccttcgcgataaggag	Fig. 2	LC269956
SiPAC-R	*PAC*	RT-PCR	ccccagctgtgtatgccaaaata	Fig. 2	LC269956
SiActin-F	*Actin*	RT-PCR	ggctggaacaagacttcagg	Fig. 2, 3	AB596843
SiActin-R	*Actin*	RT-PCR	gagccacactgttcccattt	Fig. 2, 3	AB596843
B-F	*WDR*	genomic PCR	gctcgagtagttcaaccgattt	Fig. 2	LC275141
B-R	*WDR*	genomic PCR	tgtaaaatgcactattccttgc	Fig. 2, Fig. S1	LC275141
WDR40 Walk R1st	*WDR promoter sequence*	genomic PCR	tagcttccgatggcgatggt	Fig. 2	
WDR40 Walk R2nd	*WDR promoter sequence*	genomic PCR	attgggtccggtgagtcata	Fig. 2	
1st Primer-F aroundA	*WDR promoter sequence*	genomic PCR	gtttttcgtctaccatcgatcc	Fig. 2	
1st Primer-R aroundA	*WDR promoter sequence*	genomic PCR	tttgccctaattggtttctttc	Fig. 2	
2nd Primer-R aroundA	*WDR promoter sequence*	genomic PCR	ccaatttaatccgacccagt	Fig. 2	
WDR white specific-R	*mutated WDR*	genomic PCR	gccatttttgctttcgactc	Fig. 2, Fig. S1	
A-F	*WDR*	genomic PCR	tgaaaataatgaggtgacttgagc	Fig. S1	LC275141
SiF3H full-F	*flavanone 3-hydroxylase*	genomic PCR	atggcaaaaacactgacagaac	Fig. 3	LC2269962
SiF3H full-R	*flavanone 3-hydroxylase*	genomic PCR	tcaagcaagaatctcttctatgc	Fig. 3	LC2269962
